# Coagulation/Complement Activation and Cerebral Hypoperfusion in Relapsing-Remitting Multiple Sclerosis

**DOI:** 10.3389/fimmu.2020.548604

**Published:** 2020-10-27

**Authors:** Tatiana Koudriavtseva, Annunziata Stefanile, Marco Fiorelli, Caterina Lapucci, Svetlana Lorenzano, Silvana Zannino, Laura Conti, Giovanna D’Agosto, Fulvia Pimpinelli, Enea Gino Di Domenico, Chiara Mandoj, Diana Giannarelli, Sara Donzelli, Giovanni Blandino, Marco Salvetti, Matilde Inglese

**Affiliations:** ^1^ Department of Clinical Experimental Oncology, IRCCS Regina Elena National Cancer Institute, Rome, Italy; ^2^ Department of Human Neurosciences, Sapienza University of Rome, Rome, Italy; ^3^ Department of Neuroscience, Rehabilitation, Ophthalmology, Genetics, Maternal and Child Health (DINOGMI), University of Genoa, Genoa, Italy; ^4^ Clinical Pathology and Microbiology Unit, IRCC San Gallicano Institute, Rome, Italy; ^5^ Biostatistics, Scientific Direction, IRCCS Regina Elena National Cancer Institute, Rome, Italy; ^6^ Oncogenomic and Epigenetic Unit, IRCCS Regina Elena National Cancer Institute, Rome, Italy; ^7^ Department of Neuroscience Mental Health and Sensory Organs (NEMOS), Sapienza University, Sant’Andrea Hospital, Rome, Italy; ^8^ Department of Neurology, Radiology and Neuroscience, Icahn School of Medicine at Mount Sinai, New York, NY, United States

**Keywords:** multiple sclerosis, coagulation, complement, platelets, relapse, infection, cerebral hypoperfusion

## Abstract

**Introduction:**

Multiple sclerosis (MS) is a demyelinating disease of the central nervous system with an underlying immune-mediated and inflammatory pathogenesis. Innate immunity, in addition to the adaptive immune system, plays a relevant role in MS pathogenesis. It represents the immediate non-specific defense against infections through the intrinsic effector mechanism “immunothrombosis” linking inflammation and coagulation. Moreover, decreased cerebral blood volume (CBV), cerebral blood flow (CBF), and prolonged mean transit time (MTT) have been widely demonstrated by MRI in MS patients. We hypothesized that coagulation/complement and platelet activation during MS relapse, likely during viral infections, could be related to CBF decrease. Our specific aims are to evaluate whether there are differences in serum/plasma levels of coagulation/complement factors between relapsing-remitting (RR) MS patients (RRMS) in relapse and those in remission and healthy controls as well as to assess whether brain hemodynamic changes detected by MRI occur in relapse compared with remission. This will allow us to correlate coagulation status with perfusion and demographic/clinical features in MS patients.

**Materials and Methods:**

This is a multi-center, prospective, controlled study. RRMS patients (1° group: 30 patients in relapse; 2° group: 30 patients in remission) and age/sex-matched controls (3° group: 30 subjects) will be enrolled in the study. Patients and controls will be tested for either coagulation/complement (C3, C4, C4a, C9, PT, aPTT, fibrinogen, factor II, VIII, and X, D-dimer, antithrombin, protein C, protein S, von-Willebrand factor), soluble markers of endothelial damage (thrombomodulin, Endothelial Protein C Receptor), antiphospholipid antibodies, lupus anticoagulant, complete blood count, viral serological assays, or microRNA microarray. Patients will undergo dynamic susceptibility contrast-enhanced MRI using a 3.0-T scanner to evaluate CBF, CBV, MTT, lesion number, and volume.

**Statistical Analysis:**

ANOVA and unpaired t-tests will be used. The level of significance was set at p ≤ 0.05.

**Discussion:**

Identifying a link between activation of coagulation/complement system and cerebral hypoperfusion could improve the identification of novel molecular and/or imaging biomarkers and targets, leading to the development of new effective therapeutic strategies in MS.

**Clinical Trial Registration:**

Clinicaltrials.gov, identifier NCT04380220.

## Introduction

Multiple sclerosis (MS) is a chronic demyelinating and degenerative disease of the central nervous system (CNS) with an underlying immune-mediated and inflammatory pathogenesis (1). For many years, self-reactive T cells have been considered to have an exclusive role in the pathogenesis of MS, mainly due to findings from animal disease models such as that on experimental autoimmune encephalomyelitis (EAE) (2). Recent evidence has pointed out the crucial role of both B cells and innate immunity (3–6). In particular, it is hypothesized that innate immunity, which can be considered the immediate non-specific defense against any *noxa patogena* including infections and dangerous agents, not only stimulates and modulates adaptive immunity at MS onset but also mediates its neurodegenerative progressive phase (5, 6). Both inflammation and coagulation are the main effector processes of innate immunity acting synergistically through mutual regulation (7–10). Unlike what is commonly thought, direct and functional vascular injury, such as that caused by hypoxia, sepsis, malignancy, and inflammation, can activate coagulation (11, 12). Thus, thrombosis represents a physiological process in some conditions, “immunothrombosis,” an intrinsic effector mechanism of innate immunity (13). Immunothrombosis is aimed at recognizing the pathogens and counteracting their tissue invasion, dissemination, or survival, and it should be limited to only a restricted microvascular area to ensure sufficient overall organ perfusion. It is activated by blood-borne pathogens as well as by circulating self-components that are altered, and through its action on a platform consisting of fibrin, monocytes, neutrophils, and dendritic cells lead to fibrin formation and platelet activation.

In fact, many decades ago a relevant role of both coagulation and vascular thrombosis was hypothesized in MS (14). Subsequently, a number of studies focused on the role of either thrombin, fibrin(ogen), or other coagulation factors in MS due to the findings of both a close association between perivascular fibrin(ogen) deposition and clinical manifestations in EAE and its prompt improvement after the inhibition of thrombin generation by heparin and several anticoagulant agents (15). The discovery of some clotting factors in chronic active MS lesions by a proteomic approach has further strengthened this line of investigation (16).

Fibrinogen, produced by hepatocytes and cleaved off by thrombin, is an acute-phase reactant that increases during the inflammatory response and leads to the formation of insoluble and stable fibrin facilitating the formation of a platelet plug (17–19). In a recent study, high plasma fibrinogen levels resulted in a high specificity but a low sensitivity for detection of active lesions on MRI during relapses, suggesting a role of fibrinogen in the development of MS lesions (20). Moreover, fibrinogen transcripts were found to be present in chronic lesions of MS patients (21). Fibrinogen directly activated microglia *in vitro* and increased its phagocytic ability (22). Fibrinogen also induced the release of reactive oxygen species (ROS) in microglia, necessary for the formation of perivascular microglial clusters and axonal damage in EAE (23), stimulating the production of both tissue factor (TF) (24) and tumor necrosis factor (25) by monocytes. The conversion of fibrinogen to insoluble fibrin is fundamental for the binding of fibrin to the integrin receptor CD11b/CD18 expressed by microglia (18), leading to an increase of several cytokines that modulate cell adhesion and migration (26). Fibrin deposition in MS may precede and coincide with the formation of demyelinating lesions (27, 28) and with the area of axonal damage (29). In fact, a little deposition of extravascular fibrin has been observed in chronic, non-active MS lesions as a consequence of persistent blood-brain barrier (BBB) damage (28). Finally, fibrin-targeting monoclonal antibody immunotherapy could inhibit autoimmunity without suppressing innate immunity or interfering with coagulation (30).

Furthermore, significantly higher plasma levels of prothrombin and factor X have been found in relapsing-remitting (RR) MS (31). Of note, relapse-free time negatively correlated with levels of prothrombin, factor XII, or factor X, indicating that disease exacerbation is characterized by increased coagulation activity (31). Interestingly, the speed of thrombin generation was higher in relapsing-remitting than in primary progressive MS or healthy controls and correlated with time from clinical diagnosis, likely suggesting a differential active proinflammatory state in each MS subtype (32). By the proteomics approach, some serum proteins such as anti-thrombin, ceruloplasmin, clusterin, apolipoprotein E, and complement C3 were differently expressed in RRMS patients compared to controls (33). Besides, anti-thrombin was oxidatively modified in relapse compared with remission.

Protein C (PC) is a vitamin K–dependent zymogen of a serine protease activated by thrombin when both bind to endothelial cell thrombomodulin (TM) (34). PC also binds to the endothelial protein C receptor (EPCR). Activated PC (APC) is a natural anticoagulant and with its cell membrane localizing cofactor, protein S (PS), binds to both endothelium and activated platelet membranes and interferes with the degradation of procoagulant factor Va and VIIIa, thus limiting further thrombin formation. Recombinant TM ameliorated EAE clinically and pathologically by suppressing plasma levels of inflammatory cytokines (35).

Moreover, APC contributes to endothelial cell integrity (36), inhibits leukocyte adhesion and BBB crossing (37), reduces the production of pro-inflammatory cytokines (36, 38–41), and has anti-oxidant properties (42). A potential role of APC in MS pathogenesis has been hypothesized (43) since it was found reduced in MS patients regardless of their lupus-anticoagulant (LA) activity or factor Va resistance (44).

There are conflicting results in MS regarding the role of antiphospholipid antibodies (APLs), markers of increased coagulation activity, mostly due to methodological issues and to the type of antibodies used in the assays (45). Recently, a consensus has been reached among experts that APL reactivity is higher in MS than in healthy controls. However, this finding could be variable depending on the different disease forms and phases. In particular, a higher APL reactivity appeared to be associated with a more severe clinical and MRI disease progression (46), and with clinical exacerbations, sometimes followed by its decrease in the next months after the relapse (47–50). These thrombogenic mechanisms seem to correlate with neurodegenerative processes (51) enough to consider APLs as a new attractive therapeutic target in MS for use, for example, of hydroxychloroquine, an anti-infective, anti-inflammatory, and anti-thrombotic drug with specific protective property for annexin-V anticoagulant shield (52).

There are a number of studies confirming the involvement of complement in the pathogenesis of MS, highlighting its important role due to interrelation with coagulation as well as with both innate and adaptive immunity (53–57). Its components have been proposed as biomarkers of both MS disease activity and patient therapeutic response.

Ingram and collaborators have demonstrated augmented plasma levels of either C3, C4, C4a components, C1 inhibitor, or factor H as well as reduced levels of C9 in MS patients compared with controls (55). Based on the correlations between their plasma and cerebrospinal concentrations, synthesis of these components was suggested to be localized both systemically and intrathecally. A derived statistical model combined this complement profiling with patient demographic data reaching a predictive value of 97% for MS diagnosis and 73% for clinical exacerbation.

Moreover, an immunohistochemical analysis identified the reactivity for complement proteins (C3, factor B, C1q), activation products (C3b, iC3b, C4d, terminal complement complex), and regulators (factor H, C1-inhibitor, clusterin) within and around MS lesions even in the absence of evident ongoing inflammation (56). Complement staining was also present in normal-appearing white matter (NAWM) and cortex of MS patients, albeit to a lesser extent than in MS plaques, indicating its persistent local synthesis, activation, and regulation. Reactive astrocytes, frequently adjoining to both microglia clusters and damaged myelin/axons, were largely positive for cellular complement staining. This suggests a role of complement in the pathogenesis of cell, axon, and myelin damage.

As a part of innate immunity, platelets play a relevant role in MS pathogenesis (58–62). They increase both BBB permeability and CNS inflammation by either releasing proinflammatory mediators (matrix metalloproteinases, chemokines, and adhesion molecules), displaying inflammatory molecules on their surface, or interacting with endothelial cells and leukocytes, thus, triggering the latter to infiltrate the CNS (63). Alpha-granules are the most abundant platelet secretory granules. They contain numerous soluble factors involved in coagulation such as prothrombin, TF, high molecular weight kininogen, chemokines, proangiogenic and antiangiogenic proteins, growth factors, vWF, fibrinogen, and inhibitory proteases including antithrombin III, protein S, plasminogen, and TF pathway inhibitor.

Platelets participate in the acute phase of the inflammatory response in MS by producing significant amounts of IL-1alpha and other bioactive mediators that activate brain endothelium and promote the recruitment of leukocytes triggering and amplifying cerebrovascular inflammation and brain injury (64). Moreover, platelet-activating factor receptors are up-regulated in MS lesions, and abundant platelets have been shown within the CNS inflamed area of MS patients (62).

Through the production of ROS, activated platelets represent an additional source of oxidative stress for the CNS that has antioxidant mechanisms (65). Oxidative stress is dramatically increased during neuroinflammation, leading to damage of several cellular structures, particularly myelin.

Degenerative disorders, including MS, are associated with platelet dysregulation and excessive release of extracellular vesicles containing RNA and miRNA (short single-strand sequences of non-coding RNA) constituting approximately 70–90% of all vesicles circulating in the blood (66). Among nine exosomal miRNA profiles identified as promising candidate biomarkers to distinguish relapsing-remitting from progressive MS, two platelet-enriched miRNAs, miR-30b-5p and miR-223, are drivers of platelet production (67). Moreover, various platelet-related miRNAs have been found to be associated with both MS activity and duration, and the platelet-enriched geromiR miR-155 seems to be up-regulated in MS patients contributing to MS-associated inflammation and neurodegeneration (68).

Finally, in the experimental settings, platelets have been demonstrated to be a potential therapeutic target since platelet depletion ameliorates the EAE course (69).

Neuroimaging studies have been fundamental for providing a better insight into the pathophysiology of MS. In particular, studies using quantitative contrast-enhanced MRI showed a BBB leakage of small extent in non-enhancing MS lesions, which was not influenced by ongoing therapies and was different from an evident BBB damage of enhancing lesions, likely as a result of persisting reparative thickening of vessels within chronic MS lesions (70). These BBB abnormalities prevalently reflecting alterations of “tight” junctions (TJ) were demonstrated in NAWM (71) and even in the overall vascular CNS system, the latter probably due to the systemic effect of soluble pro-inflammatory mediators (72). It is not surprising that the chronic subtle BBB breakage can determine a persistent although soft discharge of inflammatory mediators and cells from blood to CNS with a slight but lasting fibrinogen leakage (73). Fibrinogen was found to be associated with both astrocyte processes and TJ abnormalities and correlated with diffuse microglial activation and weakened axonal and myelin integrity.

Moreover, dynamic-susceptibility contrast-enhanced (DSC) perfusion MRI showed delayed cerebral blood mean transit time (MTT) and reduced cerebral blood flow (CBF) in both NAWM and NA gray matter (NAGM) either in clinically isolated syndrome or in all forms of MS. These observations support a continuum of matter perfusion deceleration initiated in WM and spreading to GM (74–76). It is conceivable that the global hypoperfusion in GM and WM of MS patients may be determined by overall blood flow deceleration due to the inflammatory-thrombotic processes, which occur physiologically in the venous vessel bed, particularly during relapses frequently associated with the recurrent infections (77, 78).

Systemic infections can cause CNS damage so much so that peripheral inflammation resulted in being associated with disease exacerbations in experimental models of both MS and other neurodegenerative diseases (79, 80). Systemic immune activation influences local innate immunity, which, in turn, conditions adaptive immune response. During acute/subacute, thus delimited, CNS damage, neuroinflammation could be resolved and concluded with a regeneration area surrounding neurodegeneration (81). Conversely, chronic neuroinflammation inevitably leads to widespread neurodegeneration that, in the same way, spreads neuroinflammation and reduces CNS regenerative capacity. Natural regeneration in injured CNS tissue is insufficient in MS due to excessive extension of neuroinflammation and neurodegeneration.

Among the likely causes of acute and chronic neuroinflammation, there are recurrent and chronic infections accompanied by physiological immunothrombosis, as reported above (13). A continuous and close inter-correlated crosstalk between immune cells and coagulation is fundamental for an effective immune response aiming to restrain the dissemination of pathogens and to potentiate their elimination and tissue repair (12). Coagulation is activated during viral infections and plays multiple functions in the host immune system (82). The recent coronavirus (COVID-19) pandemic has confirmed a relevant role of coagulation activation during viral infection, especially in severe cases, with markedly elevated D-dimer and fibrin degradation product (83).

Temporal virus-BBB interlinkage during viral infections likely determines BBB breakdown triggering neuroinflammation and demyelination (84). A large Danish nationwide case-control study found that children who developed MS have had more infections than their peers 3 years prior, likely depending on their immune reaction to infections (85). Actually, some viruses have been identified to be responsible for the disease development by causing immune activation such as Epstein Barr virus, human herpesvirus 6, Torque teno virus, varicella zoster virus, poliovirus, Picornaviridae family including rhinovirus and enterovirus, coronavirus, adenovirus, influenza virus, and respiratory syncytial virus (86). For example, high anti-HHV-6 IgG titers indicative of HHV-6 infection as well as the immune response to HHV-6 antigens influenced the risk of MS relapses and likely MS progression (87).

Indeed, infections contribute not only to MS pathogenesis but also to disease exacerbation. It seems that about 30–40% of relapses occur after an upper-respiratory infection (88); these data are also confirmed by disease activity at MRI (89). Relapse rates are positively associated with upper-respiratory infections, and approximately two upper respiratory infections per year doubles the risk for MS relapse. After a peak in diagnosed influenza A cases in the general population, it was observed that the occurrence of MS relapse was 6.5 times more likely to occur (90).

In addition, a Cochrane review concluded that some pathogens such as human herpesvirus 6, Chlamydia pneumoniae, and Torque teno virus could contribute to MS progression (91). Therefore, not only viruses but also bacteria (e.g., *Chlamydia pneumonia*, *Staphylococcus aureus*, enterotoxin A) and fungal infections seem to have a role in both MS pathogenesis and course (92).

Altogether, these studies have pointed out the role of the coagulation pathway in close correlation with infections in the MS pathophysiology and its association with brain perfusion deceleration. This suggests possible therapeutic targets that may complement existing treatments. The aim of our study is to validate the pathogenetic role of coagulation together with brain hemodynamic abnormalities in MS.

## Methods and Analysis

### Design

**Figure 1:**
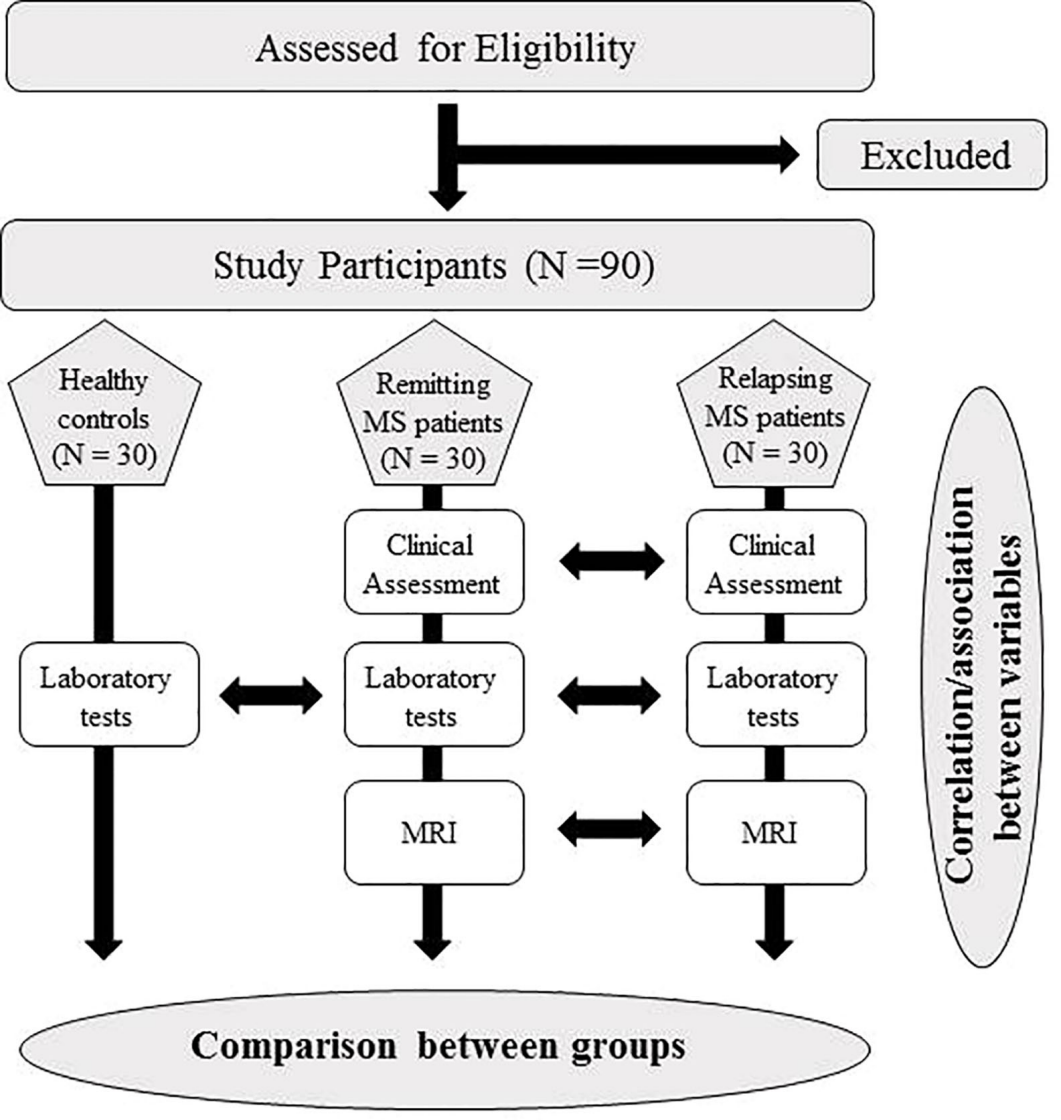
Flow chart of the study protocol.

This is a multi-center, prospective, controlled study (**Figure 1**). The study has been approved by the Ethics Committee of the IRCCS Regina Elena National Cancer Institute and by the Ethics Committee of the Sapienza University of Rome.

Specific aims of our study are

**Primary outcomes:**

- To evaluate serum/plasma concentrations of complement (C3, C4, C4a, and C9), Fibrinogen, Factor VIII (FVIII), Factor X (FX), D-dimer (DD), PC, PS in relapsing-remitting MS (RRMS) patients and healthy people.

**Key secondary outcomes:**

- To evaluate serum/plasma concentrations of Prothrombin Time (PT), Activated Partial Thromboplastin Time (aPTT), Factor II (FII), antithrombin III (ATIII), von Willebrand factor (vWF), soluble (s)TM and soluble (s)EPCR, Angiopoietin-1, Angiopoietin-2, FIII or TF, TM, Tie-2, Vascular Endothelial Growth Factor (VEGF), APLs, lupus anticoagulant (LA), complete blood count (CBC), viral serological assays, and microRNA microarray in relapsing-remitting MS (RRMS) patients and healthy people.

**Additional secondary outcomes:**

- To assess relative CBF, CBV, and MTT by DSC 3.0-T MRI in relapsing MS patients compared to remitting MS patients.
- To evaluate number and volume of enhancing lesions in relapsing MS patients compared to remitting MS patients.
- To evaluate the relationships between laboratory data, demographic/clinical (age, gender, disability and disease duration) features, and MRI perfusion findings in the patients’ groups.

### Selection of Subjects

Subjects of both genders will be recruited in two centers: the Multiple Sclerosis Center of the Sapienza University of Rome and the Multiple Sclerosis Center of the IRCCS Regina Elena National Cancer Institute. The planned recruitment period will be of three years.

Prior to enrollment, all participants will be screened to check their inclusion and exclusion criteria.

Patient inclusion criteria will be:

- Patients diagnosed with relapsing-remitting MS (93)
- Patients untreated or treated with only immunomodulatory therapy
- 18–60 years old

Patient exclusion criteria will be:

- pregnancy
- co-existing neoplastic, hematologic, thyroid, metabolic, thrombotic, or autoimmune diseases
- drug or alcohol addicted
- therapy with immunosuppressive drugs, steroids, or any medication interfering with coagulation

We will evaluate 3 groups of subjects: 30 RRMS patients in relapse (Group I); 30 RRMS patients in remission, i.e., without relapse in the previous 2 months (Group II), and 30 sex- and age-matched healthy controls (Group III).

A relapse or exacerbation or “attack” was defined by the multiple sclerosis guidelines as a manifestation of new or worsened neurological symptoms lasting for more than 24 hours (94). Symptoms should be supported by subjective description and by objective clinical assessment with no other explanation for them. The relapse is separated from the previous “attack” by at least 30 days and usually persists for days or weeks, then slowly improves over weeks or a few months ending with partial or complete recovery, i.e., remission. MRI showed the new and/or active lesions in the majority of patients, and steroid therapy often accelerates the recovery of symptoms.

Relapse needs to be distinguished from a pseudo-exacerbation, which is defined as a temporary worsening of pre-existing symptoms without new MS-related neuroinflammation, thus an MRI does not detect active or new lesions (95). The causes of pseudo-relapses could be different and include infections with or without fever, increased body temperature due to over-exercising, sauna, hot shower/bath or weather, dehydration, hormonal changes during menstruation or severe psychological stress, surgery, trauma, medications, alcohol overuse, and several medical conditions as thyroid or other metabolic disorders. Pseudo-relapses usually last less than 24 hours and resolve after removing their triggering causes with no need for steroid therapy.

We will only include MS patients in relapse who have new symptoms for more than a day but no more than a month and possibly as soon as they communicate the neurological symptoms to their doctor to quickly perform all protocol procedures before starting treatment with steroids. Patients in relapse already on steroid therapy cannot be enrolled in the study. This includes both naïve and treated patients as they could raise some concerns regarding the possibility that treatment could affect levels of some of the biomarkers studied. Indeed, it would be ideal to recruit only untreated patients, although it should be taken into account that other concomitant factors (lifestyle, nutrition, current infections, physical activity, smoking, etc.) could influence these biomarkers, albeit to a lesser extent. The choice to include patients on immunomodulating (but not immunosuppressive) therapy was dictated by the actual difficulty in finding untreated patients out of those just diagnosed. However, since the two compared groups of patients, i.e., in relapse and in remission, are recruited from the same outpatient population, the treatment influence could be sufficiently balanced between these groups and, in any case, it will be weighed in the final analysis.

An informed written consent will be obtained by all participants. The study information will be provided to the patients by the investigator to explain the study aims and protocol procedures, risks, and benefits (96). We will encourage the patients to take time before signing the consent form to discuss their participation in the study with their trusted advisors. We want to highlight that the timing of our study procedures largely coincides with the routine clinical MS management. In fact, relapsing patients usually perform blood sampling to evaluate the general clinical status and Gd-enhanced MRIs to define the extent of radiological disease activity. Regarding remitting MS patients, study participation will be proposed only to patients who need to perform their routine periodic blood tests or neuroimaging. A greater amount of blood required for additional laboratory tests pre-specified in the study and perfusion sequences, both of them with their relative low risk, merely represent the only difference between study procedures and equivalent routine exams. The benefits of participating in the study could consist of more rapid MRI execution not always easily accessible in routine practice.

On the other hand, healthy people, potentially research subjects, will have only blood sample collection for the pre-specified laboratory assessment; they will not undergo Gd-enhanced MRIs since it represents, in any case, a reasonable low risk related to the use of contrast. Patients will be seen and assessed for physical disabilities using the Expanded Disability Status Scale (EDSS) (97) and Multiple Sclerosis Functional Composite (MSFC) (98).

Each subject included in the study will remain anonymous for privacy reasons and identified by a progressive numeric code (ID number) (associated with their own name, surname, and date of birth in a separate database) so that all serum/plasma samples and MRI data will be treated and processed blindly. An ad-hoc secure database has been established to collect standardized data from different centers, data storage, and sensitive data protection.

### Interventional Methods

#### Laboratory Procedures

At enrollment, blood samples will be obtained from each participant. For most laboratory markers that will be investigated in this study (e.g., blood count test, coagulation factors, complement components, anti-cardiolipin antibody, anti-β2 glycoprotein I antibody, anti-prothrombin IgG/IgM and anti-AnnexinV IgG/IgM, RNA extraction, and Real Time polymerase chain reaction) technical procedures are already well established and are a part of the clinical laboratory practice. In regards to the technical procedures, we will use other specific molecular markers for measuring (i.e., angiopoietin-1, angiopoietin-2, FIII/TF, Tie-2, VEGF). We will refer to the available literature data and evidence.

##### Blood Processing

Plasma samples will be collected using sodium citrate as an anticoagulant at a concentration of 3.2%. Plasma and serum aliquots will be obtained within 3 hours of sample collection by centrifugation at 3,000 g for 10 minutes and 2,000 g for 10 minutes, respectively, at 25°C, and then they will be stored at -80°C up to the time of use. For specialized coagulation tests, a 20-minute double centrifugation will be performed.

##### Blood Count Test

The CBC is a routine examination of venous blood collected in tubes with EDTA anticoagulant and performed in our laboratory by automatic DxH 800 analyzer provided by the Beckman Coulter company (Brea, California).

The tool provides fast, reliable, and high-quality results thanks to Automated Intelligent Morphology (AIM) and advanced algorithms, and it avoids cellular interference with Coulter’s principle impedance and multi-angle scatter laser.

It analyzes the number of red blood cells (erythrocytes or RBC), white blood cells (leukocytes or WBC), platelets (thrombocytes), hematocrit (HCT), and hemoglobin (Hb) levels of the leukocyte formula (percentage of different white blood cells: neutrophils, lymphocytes, monocytes, eosinophils, and basophils). It determines the physical characteristics of the red blood cells by means of the following indices: MCV (average corpuscular volume), MCH (average corpuscular hemoglobin content), MCHC (Medium Corpuscular Hemoglobin Concentration), RDW (red blood cell distribution amplitude), and MPV platelets (mean platelet volume).

##### Measurement of Coagulation Factors

All routine coagulation factors such as DD, Fibrinogen, ATIII, aPTT, PT, and specialist test of coagulation as PC, PS, FII, FVIII, vWF, and LA will be performed by ACL TOP 750 analyzer. This instrument is fully automated, fast and reliable, and able to detect all coagulation, chromogenic, and immunological reactions using a photo optical detection system by HemosIL commercial kits (Instrumentation Laboratory Co.). This guarantees maximum precision and accuracy of the results with the transmission of data to the laboratory computer system.

##### Measurement of Complement Components: C3, C4, C4a, C9

Serum concentrations of complement C3 and C4 components will be evaluated with Cobas 8000 fully automated platform by Roche Diagnostics. This instrument is based on an immunoturbidimetric principle. The reference ranges include 90–180 mg/dL and 10–40 mg/dL for human C3 and C4, respectively. The sensitivity of the test is 4 mg/dL for C3 and lower than 0.02 g/L for C4.

The fragment C4-a and component C9 were separately quantified by sandwich enzyme-linked immune-sorbent assay technology (ELISA) using two different kits purchased by Nordic BioSite AB.

###### Sample Preparation

The serum samples have to be left for 2 hours at room temperature or overnight at 4°C before centrifugation for 20 minutes at about 1000 g, and the supernatant is collected and the test must be performed either immediately or stored at -80°C until use.

###### Assay Procedure

Before running the test, the reagents will be prepared and the samples will be diluted as required in the kit. In detail, the test is performed on a 96-well plate, pre-coated with anti-C4a or anti-C9 antibodies. Standards and diluted samples are added to each well and incubated at 37°C for 90 minutes followed by three incubations at 37°C by suitable washes: the first with 100 µl of biotin conjugated anti-C4a or anti-C9 antibody for 60 minutes, the second with 100 µl of HRP-Streptavidin Conjugate (SABC) working solution for 30 minutes, and the third with 90 µl of substrate (TMB) in dark for 15–30 min to visualize HRP enzymatic reaction. TMB is catalyzed by HRP and produces a blue colored product that changes to yellow after adding 50 μL acidic stop solution. The plate is read at 450 nm in a microplate reader. The density of yellow is directly proportional to C4a or C9 concentration of sample. The reference ranges are: 1.563–100 ng/mL and 0.234–15 ng/mL for human C4-a and C9, respectively. This assay has high sensitivity (< 0.938ng/ml) and excellent specificity.

##### Measurement of sEPCR

Plasma levels of sEPCR will be performed by quantitative sandwich enzyme immunoassay (ELISA) Kit (Cusabio Biotech CO., LTD.). The detection range established by the manufacturer ranges from 7.8 ng/mL to 500 ng/mL. The dosage has a sensitivity lower than 1.95 ng/mL and high specificity.

##### Measurement of Angiopoietin-1, Angiopoietin-2, FIII/TF, TM, Tie-2, VEGF

**Figure 2:**
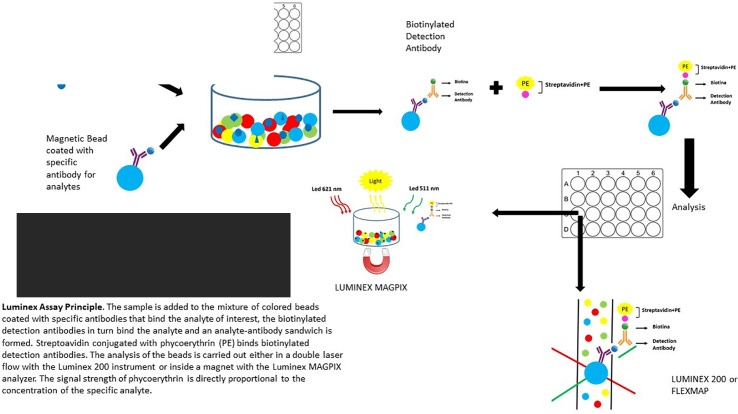
Luminex Assay Principle. The sample is added to the mixture of colored beads coated with specific antibodies that bind the analyte of interest, the biotinylated detection antibodies in turn bind the analyte, and an analyte-antibody sandwich is formed. Streptoavidin conjugated with phycoerythrin (PE) binds biotinylated detection antibodies. The analysis of the beads is carried out either in a double laser flow with the Luminex 200 instrument or inside a magnet with the Luminex MAGPIX analyzer. The signal strength of phycoerythrin is directly proportional to the concentration of the specific analyte.

Angiopoietin-1, Angiopoietin-2, TF, TM, Tie-2, and VEGF biomarkers will be performed by Magnetic Luminex Assay multiplex kits supplied by R&D Systems, Inc. (USA) (**Figure 2**).

It is a premixed multi-analyte human kit, which can be used to evaluate up to 50 human biomarkers in a single supernatant, serum, plasma, and cell culture sample. The test is based on quantification immunoassay and the innovation of this test is represented by the use of magnetic microparticles. Determined antibodies for the analytes of interest are pre-coated to each specific region of the microparticle labeled with different fluorophores.

###### Sample Preparation

Fresh and previously frozen serum or plasma samples require centrifugation at 16000 g for 4 minutes immediately before use. Samples should be diluted correctly as required by the kit.

###### Procedure Assay

The diluted microparticle cocktail will be suspended by inversion or on a shaker, then 50 µl of microparticle cocktail, 50 µl standard, and 50 µl of samples will be added to each well of the microplate and incubated for 2 hours at room temperature on a horizontal orbital shaker for microplates (0.12 Orbit) set at 800 ± 50 rpm. Using a magnetic device, three washes with 100 µl of wash buffer will be performed to eliminate unbound substances, and 50 µl of the biotinylated antibody cocktail specific will be added to the analytes of interest to each well for 1 hour at room temperature on the shaker set at 800 ± 50 rpm. The washes will be repeated and 50 µl of the streptavidin-phycoerythrin conjugate will be added (Streptavidin-PE) to each well. Then it will be incubated for 30 minutes at room temperature on the shaker set at 800 ± 50 rpm. Final washes will be performed to remove unbound Streptavidin-PE. Finally, the microparticles will be resuspended in 100 µl of Wash Buffer for 2 minutes on the shaker set at 800 ± 50 rpm and within 90 minutes the plate will be read using a Luminex^®^ MAGPIX^®^ or Bio-Rad analyzer. A magnet in the analyzer captures and holds the superparamagnetic microparticles in a monolayer. Two spectrally distinct light emitting diodes (LEDs) illuminate the microparticles. One LED excites the dyes inside each microparticle to identify the region, and the second LED excites the PE to measure the amount of analyte bound to the microparticle. A sample from each well is imaged with a CCD camera with a set of filters to differentiate excitation levels. Analysis with the Luminex^®^ 100/200™, Luminex^®^ FLEXMAP 3D^®^, or Bio-Rad Bio-Plex uses one laser to excite the dyes inside each microparticle to identify the microparticle region and the second laser to excite the PE to measure the amount of analytes bound to the microparticle. All fluorescence emissions from each microparticle as it passes through the flow cell is then analyzed to differentiate emission levels using a Photomultiplier Tube (PMT) and an Avalanche Photodiode. The assay has a higher sensitivity than the traditional ELISA and requires a smaller sample volume, which is fast and efficient.

##### Measurement of APLs

###### Measurement of Anti-Cardilipin (aCL) and Anti-β2 Glycoprotein I (aβ2GPI)

The semi-quantitative detection of aCL and aβ2GPI of both IgG and IgM classes will be evaluated in serum by Kit BioPlex 2200 System Antiphospholipid Syndrome (APS) supplied by BIO-RAD Laboratories, Marnes-la-Coquette, France. The test is based on the multiplex flow immunoassay that uses microspheres coated with aCL and aβ2GPI antigens. It is a fully automated test.

*Procedure assay:* The test requires two incubations steps at 37°C into a unique reaction vessel, separated by washing: in the first step the dyed microspheres are added to the diluted sample, and in the second step the IgG and IgM antibodies conjugated to phycoerythrin (PE) and added in the reaction vessel. The microspheres mixture passes in laminar flow and is analyzed by the detector with two lasers.

Signals from a laser classify the microspheres, while those from another laser measure the fluorescence of the conjugate. The system software converts the conjugate signal into a fluorescence value (RFI). The values of specific antibodies are established by the manufacturer and expressed in GPL/MPL (aCL) and U/mL (anti-Beta2GPI). Samples with values of aCL or aβ2GPI (either IgG or IgM) inferior to 20 are evaluated as negative, values ≥ 20 to 40 weakly positive, values ≥ 40 to 80 average positive, and values ≥ 80 to 160 strongly positive. The sensitivity and the specificity of aCL and aβ2GPI (IgG) test is 67.5% and 100%, respectively. The sensitivity and the specificity of aCL and aβ2GPI (IgM) test is 25.8% and 98.7%, respectively.

###### Measurement of Anti-Prothrombin IgG/IgM and Anti-AnnexinV IgG/IgM

The quantitative measurements of IgG/IgM class autoantibodies against AnnexinV and Prothrombin in the serum will be determined by an indirect enzyme linked immune reaction (ELISA) kit supplied by DRG International, Inc., USA. The measurement range of this ELISA test was established by the manufacturer for Anti-AnnexinV and Anti-Prothrombin IgG/IgM ranges from 0 to 100 U/mL. The cut-off for Anti-AnnexinV and Anti-Prothrombin IgG/IgM is 8 U/mL and 10 U/mL, respectively.

Samples with values of Anti-AnnexinV IgG/IgM <5 U/mL are considered as negative, with values between 5 U/mL and 8 U/mL as borderline, with values ≥ 8 U/mL as positive. Samples with values of Anti-Prothrombin IgG/IgM <10 U/mL are considered as negative and with values ≥ 10 U/mL as positive. Limit of detection of Anti-AnnexinV and Anti-Prothrombin is of 1U/mL either IgG or IgM. The specificity of Anti-AnnexinV is 95.8% and 96.7% for IgG and IgM test, respectively. The specificity of Anti-Prothrombin is 98% and 99.3% for IgG and IgM test, respectively.

##### RNA Extraction and Hybridization on Agilent Microarray

**Figure 3:**
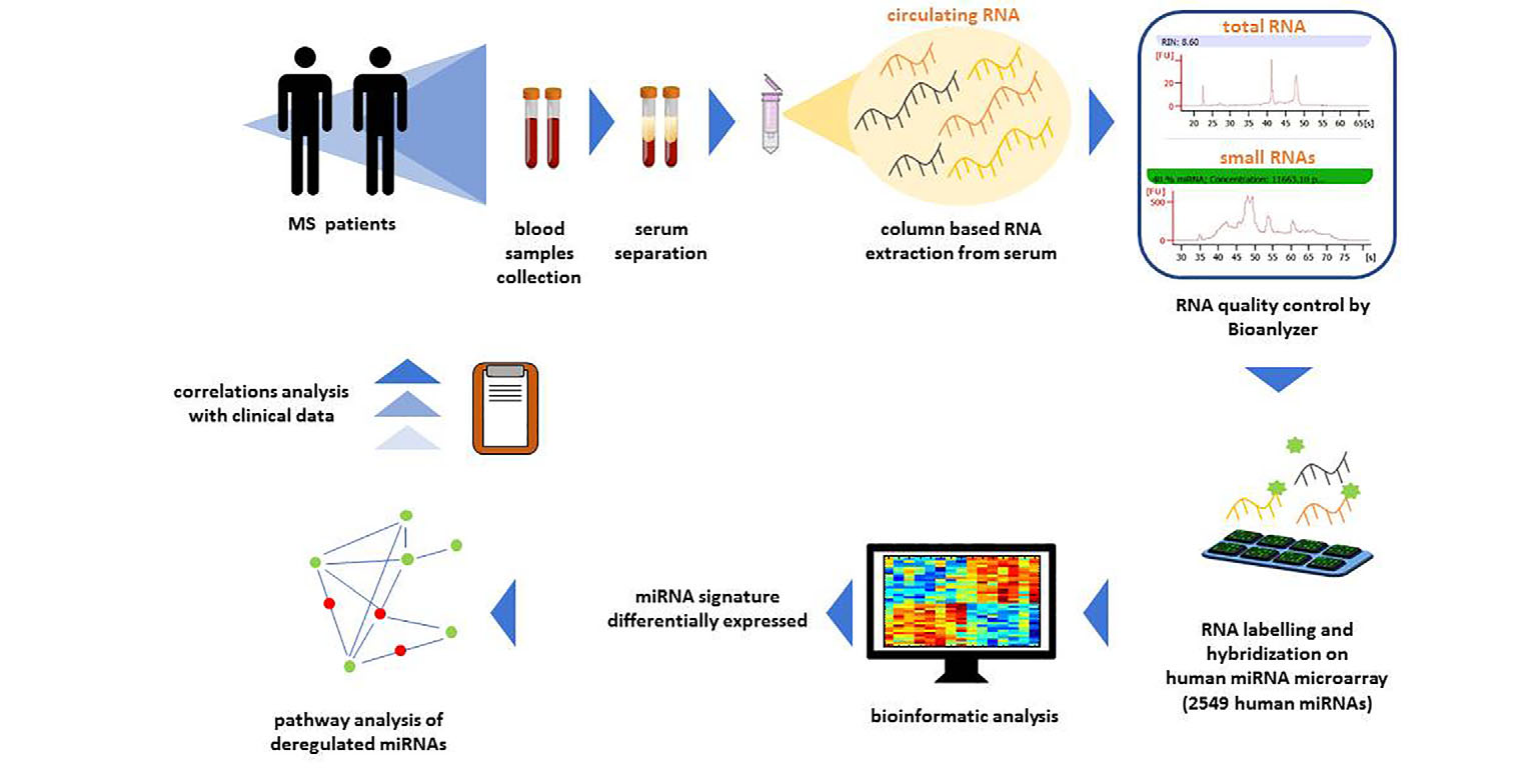
Workflow of circulating miRNA profiling. For circulating miRNA profiling blood samples from patients will be processed. In particular, circulating RNA will be extracted from serum samples with a column-based extraction method. Total and small RNA quality will be assessed by Bioanalyzer. Then, total RNA will be labelled for the hybridization to Human miRNA Microarray Release 21 (Agilent) containing probes for 2549 human miRNAs. Microarray data will be subjected to bioinformatic analysis to identify a signature of miRNAs differentially expressed. Deregulated miRNAs pathway analysis and correlations analysis with clinical variables will be performed.

Total RNA extraction from sera will be performed by a column-based method that includes small RNAs and minimizes the carry-over of enzyme inhibitors typically contained in biofluids (miRNEeasy serum/plasma kit, QIAGEN) (**Figure 3**).

Total RNA will be labeled and hybridized to Human miRNA Microarray Release 21 (Agilent) containing probes for 2549 human microRNAs from the Sanger database. Each slide is an 8 x 15K format (~15,000 features printed in an 8-plex format, eight individual microarrays on a 1” x 3” glass slide) printed using Agilent’s 60-mer Inkjet Technology, which, unlike competitive platforms, synthesizes 40–60-mer oligonucleotide probes directly on the array, resulting in high-purity, high fidelity probes. This miRNA platform requires small input amounts of total RNA—in the 100 nanogram range—because it uses a high-yield labeling method and does not require size fractionation or amplification steps that may introduce undesired bias during miRNA profiling.

Scanning and image analysis will be performed using the Agilent DNA Microarray Scanner (P/N G2565BA) equipped with extended dynamic range (XDR) software according to the Agilent miRNA Microarray System with miRNA Complete Labeling and Hyb Kit Protocol manual. Feature Extraction Software (Version 10.5) will be used for data extraction from raw microarray image files using the miRNA_105_Dec08 FE protocol. microRNA Microarray data will be subjected to stringent quality controls and then analyzed by the Bioinformatic Unit.

##### Real Time Polymerase Chain Reaction

We enable automated purification of DNA and RNA (Qiasymphony–Qiagen) from a broad range of sample types, amplifying the DNA sequences and then analyzing the products. For overcoming the challenges of limited samples and a costly analysis we will choose, when possible, Multiplex PCR that enables the amplification of more than one target in a single reaction using different reporters with distinct fluorescent spectra (Seegene Korea). Multiplex qPCR requires the use of probe-based assays, in which each probe is labeled with a unique fluorescent dye, resulting in different observed colors for each assay. The signal from each dye is used to quantitate the amount of each target separately in the same tube or well. The availability to multiplex therefore allows the measurement of the expression levels of several targets or genes of interest quickly. In particular, we will analyze the following markers by qPCR: Epstein Barr virus, human herpesvirus 6, Torque teno virus, varicella zoster virus, poliovirus, Picornaviridae family including rhinovirus and enterovirus, coronavirus, adenovirus, influenza virus, and respiratory syncytial virus, Chlamydia pneumoniae, Staphylococcus aureus, and enterotoxin A.

#### MRI Procedures

All RRMS patients will undergo the 3.0-T MRI within two weeks of enrollment. We will use DSC perfusion technique acquired during the first pass of gadolinium to estimate perfusion features inside the damaged tissue of relapsing and remitting MS patients.

DSC MR images will be acquired on the axial plane during the first pass of a standard-dose bolus (0.1 mmol/kg) of gadopentetate dimeglumine (Magnevist; Berlex Laboratories, Wayne, NJ, USA) with a gradient-echo T2-weighted echo-planar imaging sequence. A contrast will be injected at a rate of 3,5 mL/sec, followed by a 20-mL bolus of saline also at a rate of 3,5 mL/sec. A total of 60 images will be acquired at 1-sec intervals, with the injection occurring at the fifth image, for a total acquisition time of 2 min 16 s. The imaging parameters will be as follows: TR/TE = 2140/30 ms, flip angle = 30°, slice thickness = 4 mm, FOV = 280 mm, matrix = 128x128.

The following hemodynamic parameters will be obtained from the concentration–time curves: the relative CBV (rCBV, i.e., the fraction of the tissue volume occupied by blood), the relative CBF (rCBF, i.e., the volume of blood in a given amount of tissue per unit of time), and MTT (the average time it takes for the contrast agent to travel through the tissue vasculature, for the ideal case of an instantaneous bolus injection) by using NordicIce software package.

Thus, we will use the leakage correction function provided by NordicIce software to minimize this effect both on Gadolinium and on non-Gadolinium enhancing lesions, in which a subtle leakage of contrast agent cannot be excluded.

**Figure 4:**
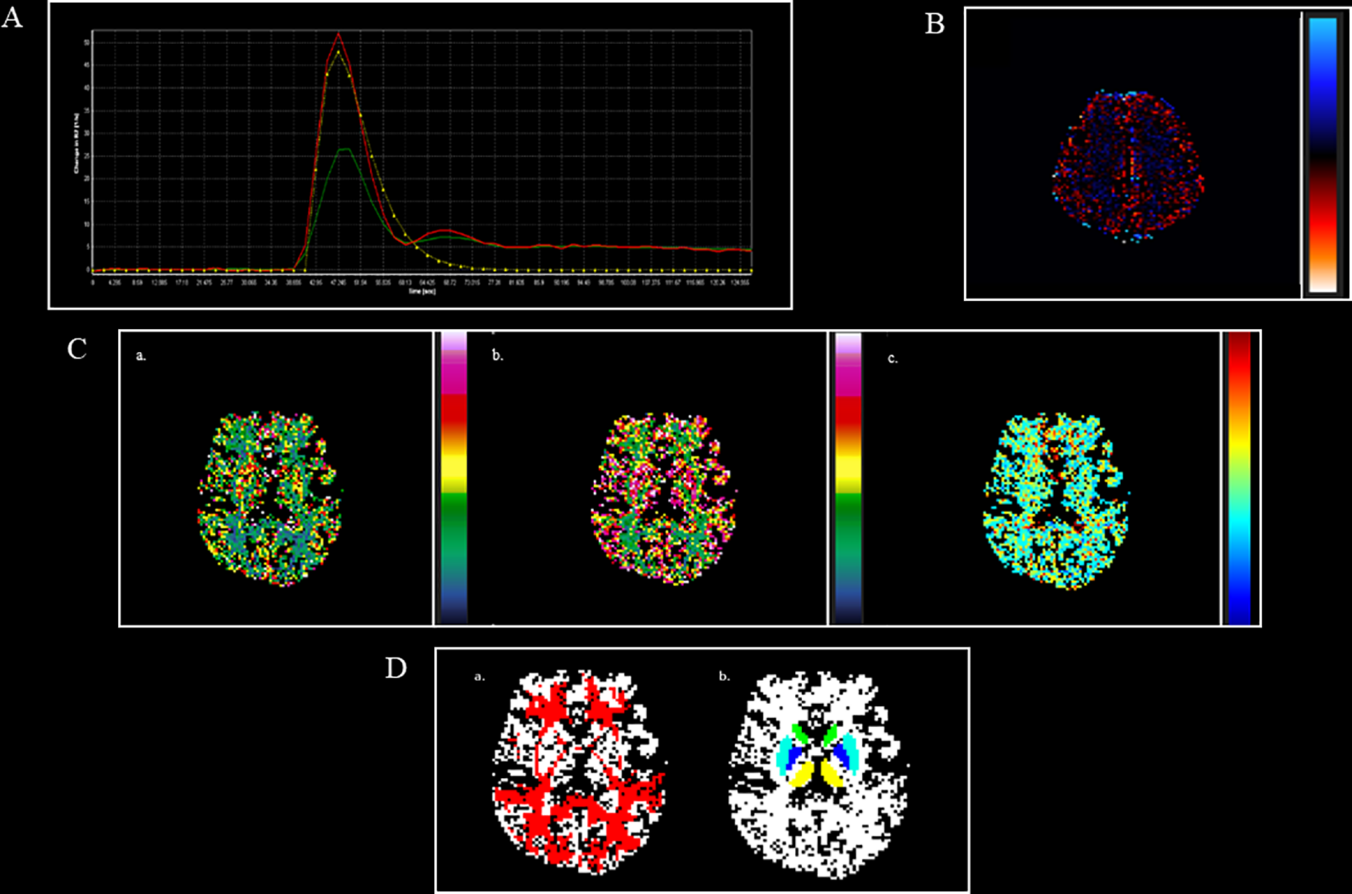
DSC perfusion maps and their overlap with structural masks in an MS patient. **(A)**. AIF curve created by using global, automatic, and outside-artery technique **(B)**. Leakage map, obtained through the leakage correction function, to minimize leakage effect both on Gadolinium and no Gadolinium enhancing lesions **(C)**. a. CBV map; b. CBF map; c. MTT map. **(D)**. a. NAWM (red), obtained by subtracting from white matter (WM binary) masks the different types of lesions, linearly registered to CBV map; b. thalamus (yellow), caudate (green), putamen (light blue), globus pallidus (blue), obtained by using FIRST software, linearly registered to CBV map. AIF, arterial input function; CBV, cerebral blood volume; CBF, cerebral blood flow; MTT, mean transit time; NAWM, normal appearing white matter.

Finally, rCBF, rCBV, and MTT values will be extracted from different sites of the brain damaged by MS (hyperintense T2-w lesions, Gadolinium enhancing T1-w lesions, periventricular and frontal NAWM, thalamus, and putamen nuclei and head of the caudate bilaterally). To exclude interobserver and to minimize intra-observer variability, each data set will be reviewed by two expert radiologists at the same time (**Figure 4**).

### Data Analysis

#### Sample Size Calculation

Overall 90 subjects (30 for each group) will be enrolled to compare the level of complement C4a (55). By using the ANOVA test, this sample size will allow detection of effect size values [delta=(miA–miB)/sigma] equal to at least 0.71, with a statistical power of 80%, to a level of significance of 5%.

#### Statistical Methods

Descriptive statistics will be used to summarize pertinent study information. Correlations between quantitative variables will be assessed with the Pearson r correlation. The associations will be analyzed by the Fisher exact test or Chi Square test for trends. Comparisons between disease subgroups and control group will be carried out for different variables, using either Student’s *t-*test or analysis of variance (ANOVA). If the ANOVA shows a statistical difference between subgroups, a post-hoc analysis with Bonferroni correction for multiple comparisons will be performed. For non-normally distributed data, non-parametric (Mann Whitney-U or Kruskal-Wallis-H) tests will be used. The level of significance is set at p ≤ 0.05 (SPSS version 20.0, SPSS Inc., Chicago, Illinois, USA).

## Discussion

While we await our results, we discuss here the methods chosen to conduct our study.

### Blood Count Test

Gens et al. examined 2.145 whole blood samples to evaluate the analytical performance between two hematology analyzers: Sysmex XN 3000 and UniCel DxH 800. For both analyzers, the variation coefficients (CV%) for hemoglobin, RBC, MCV, WBC were <5%, for WBC less than 10%, while the variation coefficients for platelets and monocytes were <5%, 15%, 6%, and 9.5%, 45%, respectively for Sysmex XN 3000 and DxH 800. The analyzers are equally precise (R>.86), with the exception of monocytes and basophils (99).

Barnes and his collaborators furthermore evaluated a better sensitivity and specificity of the DxH 800 analyzer compared to the previous Beckman Coulter LH 750 series. The DxH 800 instrument is more skilled in capturing explosions; in fact, out of 95 samples containing a burst percentage > or = 1%, the LH 750 detected 6.4% of false negatives, while the DxH 800 0.0%. The advantage of having a low false positive number has reduced peripheral blood smears for microscopic blood analysis, a time-consuming technique, and the use of an expert operator (100).

In another study, the Beckman Coulter UniCel^®^ DxH 800 analyzer was compared with the Coulter^®^ LH 780 and flow cytometry (FCM), and it was found that the DxH 800 instrument has greater sensitivity and specificity for counting WBC, PLT, and NRBC with fewer false negatives for NRBC compared to LH 780 and greater accuracy for PLT and NRBC counting than FCM (101).

### Angiopoietin-1, Angiopoietin-2, FIII/TF, TM, Tie-2, VEGF

The ELISA is the most commonly used method in both diagnostics and clinical research. However, it has some limitations. This technique requires large volumes of sample to capture an antigen of interest. In some cases, the larger surface of the wells can favor non-specific bonds, and the resulting fluorescence signal is not always linear and may invalidate the test. The assay performed on multiplex platforms also allows greater flexibility, reduced sample volume, and lower cost, with a similar workflow. Luminex xMAP technology is an array platform that allows both monoplex and multiplex assays that can be applied to either protein or nucleic acid applications. Microspheres have a smaller surface and the non-specific bond is significantly reduced (102).

Using multi-array and electrochemiluminescence technologies, the MSD platform offers multiplex capability with a consistency similar to that observed in ELISA with reduced costs and labor (103).

### Coagulation Parameters

Laboratory automation began many years ago and has since spread across other fields such as hematology, immunology, molecular biology, and coagulation tests. The advantages of automation are either standardization, error reduction, cost reduction, or productivity increase, whereas the only disadvantage is its high maintenance costs (104).

The hemostatic measurements are influenced by the technique of the instrument and the reagents used for individual analyzes (105).

Geens and his collaborators compared the performance of System CS5100 and Stago STA-R analyzers for determining routine coagulation parameters such as aPTT, PT, FBG, DD, and AT (106).

All parameters including imprecision, accuracy, and total error were deemed acceptable for the two methods; however, the difference between them consists of both high sensitivity of the CS5100 for APTT towards the deficiencies of the factors and presence of unfractionated heparin.

Unlike Stago STA-R analyzers, the CS5100 can automatically control pre-analytical variable such as sample volume and interfering substances such as hemolysis, hyperbilirubin-hememia, and lipemia but fails to analyze highly lipemic and icteric samples that represent a disadvantage in routine practice.

The APTT reagent of CS5100 showed sensitivity between 46% and 72% to FVIII, IX, XI, and XII factors while the PT reagent showed sensitivity between 34% and 52% to FII, FV, FXII, and FX factors. This explains the reference interval for APTT between the two instruments (23–31s on CS5100 vs. 30–42s on STA-R Evolution).

Furthermore, a small increase in the percentage of PT and a slight decrease in PT (INR) and FBG on the CS5100 was observed compared to the STA-R Evolution instrument (106).

Hemolysis (4%), hyperbilirubinemia (11%), and lipemia (13%) are the main preanalytical variables that cause errors in blood sample coagulation tests (107).

Woo-Jae Kwoun and coworkers assessed the performance of the pre-analytic module of the ACL TOP 750 analyzer where the reference values obtained were compared with those of the XPT instrument for chemistry, which uses an enzymatic method, and with those of ADVIA2120i, which uses a spectrophotometric method (108). The researchers concluded that an efficient control of the pre-analytical variables is exercised by the ACL TOP family series 50 spectrophotometric apparatus module ensuring sample quality monitoring, while accurate test results from the interference of the HIL sample.

The ACL-TOP analysis system produces three types of curves. The first curve shows changes in absorbance during aPTT measurement. The second one, derived from absorbance, is related to the speed of coagulation. The third one measures the acceleration of coagulation. Tokunaga and his collaborators noted the second derivative curve’s utility for detecting factor deficiencies (109). Shortcomings were found not only in FVIII but also in FIX, FXI, FXII, and FV. It has been reported that the ACL TOP system that uses the APTT-SP reagent comprising silica to be the most suitable for detecting intrinsic deficiencies of coagulation (110). Monitoring vWF on plasma from patients with acquired von Willebrand syndrome was evaluated and showed less than 10% of the activity of the vWF cofactor of both ristocetin and vWF antigen (111). After 15 minutes of desmopressin infusion (vasopressin 1-deamin-8-D-arginine; DDAVP), and based on the variations of the waveforms in an aPTT assay on the second derivative curve, levels of vWF and FVIII remarkably increased to 54% and 84%, respectively (111). These results explain that the waveform analysis of the second derivative curves of an APTT assay provide useful information relating to both reduction of coagulation factors and therapeutic treatment.

The ACL TOP instrument efficiently performs specific and routine coagulation tests for up to 120 samples simultaneously with high quality starting from smaller samples and reagent volumes rather than manual methods. Its use in many laboratories is determined by either its precision, reliability, high productivity, or daily maintenance of 4 minutes.

It is also equipped with a software capable of rerunning automatically multiple tests simultaneously using different dilutions.

The intra-assay and inter-assay precision (coefficients of variation) were less than 5% for most coagulation parameters in both the normal and pathological range (112). The results of coagulation tests obtained by the ACL TOP are well correlated with those obtained on the STAR analyzer characterized by a correlation coefficient (r) ranging from 0.876 to 0.990 (113).

#### Complement

It is necessary to standardize tests evaluating the function of the classical, alternative, or lectin pathway since the analysis of the complement system, with the exception of some proteins such as C3 and C4, varies widely between laboratories. Autoantibodies such as anti-C1q, convertases C3 and C4, or regulatory proteins like inhibitor of anti-C1, anti-factor H, are relevant in defining autoimmune processes and diseases based on complement dysregulation. The standardization committee of the International Complement Society (ICS) and the International Union of Immunological Societies (IUIS) have provided guidelines that ensure the quality of tests for the complement analysis (114).

Laboratory analyses for triggering the activation of the classical complement pathway are performed with methods based on the principles of nephelometry, turbidimetry, and ELISA (115). Complement deficiency is commonly detected in the laboratory by quantifying the main soluble fragments C3 and C4 formed during activation.

Li H. and his colleagues measured complement levels C3 and C4 in Chinese patients with systemic lupus erythematosus (SLE). The complement assay was performed based on the dispersion turbidimetry immunization rate using the Beckman Coulter instrument (Inc. Brea, CA, USA). The reference values for C3 were between 0.79 and 1.52 g/L and those for C4 were between 0.16 and 0.38 g/L, in line with those used in our laboratory measured by immunoturbidimetry and the COBAS 8000 instrument (116).

A recent study compared the levels of C3 and C4 fragments of patients diagnosed with SLE with those of healthy subjects by nephelometry. C3 and C4 values were significantly higher (p <0.001) in healthy subjects than in patients.

Sensitivity and specificity for complement C3 are 87.11% and 82.74%, respectively, and for complement C4 are 88.66% and 77.43%, respectively (117).

Myriam and his collaborators studied the activation product of plasma complement C4d of patients with SLE as a marker of lupus nephritis by ELISA. The test found significantly higher C4d values in SLE patients compared with healthy subjects, whose C4d levels were negligible.

Levels of C4d discriminated the highest and lowest clinical disease activity with a positive predictive value of 68% and a sensitivity of 79% for identifying patients with nephritis. The test could detect the lowest concentration of 5.6 μg/L. The precision of the test was demonstrated by the intra-assay and inter-assay coefficients of variation of 13.2% and 16.7%, respectively (118).

According to a previous study regarding good monitoring of C4d marker disease, the availability and ease of the test, long execution times, probable sources of error by the operator, and detectable false positives are all disadvantages.

#### Antiphospholipid Antibodies

The laboratory criteria for APS were revised and published in 2006 due to the heterogeneity of aPL, plasma proteins, or protein complexes related to them, as well as to the harmonization of the assays diagnosing the APS in order to improve the detection of aPL antibodies and the interpretation of the results (119).

The new criteria include the LAC test, aCL IgG/IgM, and aβ2GPI IgG/IgM, measured by different types of solid phase immunoassays. Currently, these immunoassays have not been entirely standardized (120).

The aPL immunological test provides information that was not obtained from the LAC test such as specific antiphospholipid analytes, isotypic class (IgM or IgG), and their concentration levels. The solid phase of the immunoassay is important and should not be influenced by analytical variables, like anticoagulant or anticoagulant therapy, as they represent interference factors for the LAC test.

In addition to the ELISA test, either chemiluminescence immunoassays (CIA), enzyme fluorescence immunoassays, or new emerging technologies such as multiple dosing through microspheres are widely used in clinical diagnostic laboratories (119). CIAs have recently been developed for the detection of aPL antibodies and are currently used in a number of clinical laboratories. CIAs are advantageous due to their extreme sensitivity and capability to be automated (121).

A number of studies have demonstrated that the performance of CIAs is similar to both commercial and laboratory developed ELISAs for aPL criteria (122).

It has recently been suggested that CIAs improve reproducibility and inter-laboratory correlations for these analytes (123).

Despite these developments, there are still no generally accepted reference reagents for the development and calibration of these tests. It is necessary to compare the performance of the new methods used for detecting aPL antibodies with the more traditional ELISA adopted to identify the commutability in the diagnosis of the APS.

Testing for aPL antibodies has traditionally been performed by ELISA due to both easy use and widespread availability (124).

Thomas B. Martins and his collaborators evaluated methods for detecting aCL and aβ2GPI antibodies in patients with APS, and concluded that the two methods are comparable; however, CIA was found to be more sensitive in detecting aβ2GPI IgG while ELISA was more sensitive to aCL IgM. Lastly, the CIA compared to ELISA method was associated with a higher number of LAC-positive APS patients.

In agreement with this, a more recent study showed a good correlation (> 80%) between the ELISA and CIA methods (125). This is also evident in the study of the Iwaniev et al., which, however, reported a significantly lower detection of IgM aCL antibodies (126).

#### MRI

We decided to use DSC perfusion, which is the most popular perfusion imaging technique applied (127), particularly due to its very fast acquisition time (approximately 1 min acquisition time), as well as the use of conventional and widely available MRI sequences (e.g., gradient-echo echo-planar imaging, EPI), and its very good contrast-to-noise ratio compared with other perfusion imaging methods, such as ASL and Dynamic contrast-enhanced (DCE). The DSC technique relies on drop in the T2 signal after passing a gadolinium‐based contrast agent (128).

Indeed, when the contrast agent reaches the vessels, it makes them more paramagnetic, and field inhomogeneities around the vessels are created. Thus, the concentration of the contrast agent may be derived from the loss in the signal intensity–time curve due to susceptibility effects of the contrast agent itself.

DSC perfusion may be obtained by using both gradient-echo and spin-echo sequences, which uses a spin-echo-planar scan. On the gradient-echo sequence, the effect of a contrast agent is stronger compared to the spin-echo signal due to the fact that the former has an additional static dephasing of spins in the same inhomogeneous environment. Different studies demonstrated that sensitivity of gradient-echo DSC is similar for a broad range of vessel sizes while spin-echo DSC is particularly sensitive to capillary-sized vessels (129).

A key role in quantifying CBF by using this technique is played by the so-called arterial input function (AIF), which describes the contrast agent input to the tissue of interest. Due to its fundamental role, many studies in recent years have focused on how and where to measure the AIF (global or regional, inside or outside the artery, manually or automatically), how DSC-MRI quantification may be influenced by AIF determination, what artefacts may be related to it, and the design of automatic processes to measure the AIF (127) In this study, we decided to perform a global, automatic, and outside-artery determination of AIF (127).

One of the main limitations of the DCS perfusion technique relies on the possible extravasation of the contrast agent due to the damage of BBB that may lead to T1 and T2* relaxation effects and, thus, to underestimating or overestimating rCBV, respectively (127). These leakage effects may partially be corrected by using a preload contrast bolus OR leakage correction algorithm.

## Conclusion

In this study, it will be important to identify the exact links between activation of coagulation/complement system and brain hemodynamic changes with cerebral hypoperfusion.

We hypothesized that cerebral hypoperfusion in all forms of MS could be the result of the blood flow deceleration mostly in the venous vessel bed during brain inflammatory-thrombotic processes in the course of relapses. Systemic immune activation during the infections influences innate brain immunity and, consequently, adaptive immune response (79, 80). When recurrent and chronic infections, which manifest systemically with immunothrombosis (13), directly or indirectly involve the CNS, it could lead to acute and chronic neuroinflammation. Constant crosstalks between immune cells and coagulation are seminal for an effective immune response (12). While many efforts have been carried out to better define the function of innate immune cells in order to modulate their potential pathogenetic role in MS with specific therapeutic action (5, 6), the coagulant component of innate immunity, which is well studied in animals, has not been sufficiently evaluated in humans. Our working hypothesis is that relapsing patients could have a pro-coagulant condition that may be correlated with blood flow deceleration and the presence of serological indicators of ongoing infection. Whether or not we found correlations between laboratory and MRI parameters, we may see the difference between the relapsing and the remitting MS patient groups. One could argue that peripheral laboratory parameters measured in this study may not fully reflect nor be specific for pathophysiological events occurring in the CNS. However, we hypothesize and believe that events at the CNS level, particularly in MS, could partially represent or be a result of systemic diseases such as infections.

Even if the activation of the coagulation system linked to innate immunity is a mandatory process following different types of tissue damage, interfering with the coagulation system could represent a new therapeutic target in MS. This approach may lead to improved treatment options (e.g., polytherapy) and the development of new therapeutic perspectives for MS and demyelinating diseases in general, but also for other neurodegenerative conditions. It is already possible to interfere with the coagulation system at various levels of the cascade. Therefore, clinical trials trying to transfer the promising results on EAE to humans are needed.

## Ethics Statement

The studies involving human participants were reviewed and approved by Local ethical committee of IRCCS Regina Elena National Cancer Institute, Rome, Italy. The patients/participants provided their written informed consent to participate in this study.

## Author Contributions 

TK and MI devised the project, the main conceptual ideas, and proof outline. TK designed and directed the project. AS, MF, CL, CM, LC, GD, FP, SZ, SD, and GB worked out almost all of the technical details. DG performed the numerical calculations for the suggested experiment. TK and AS wrote the manuscript with input from all authors. CL, SL, FP, SD, MI, MF, MS, and ED contributed to the writing of the manuscript. AS, SD, and CL designed the figures. All authors contributed to the article and approved the submitted version.

## Funding

This study is funded by the Italian Ministry of Health (Project code: PE-2013-02357745).

## Conflict of Interest

TK received a grant from the Italian Ministry of Health. MI received grants from the National Institutes of Health, National Multiple Sclerosis Society, and FISM and received fees for consultation from Roche, Genzyme, Merck, Biogen, and Novartis. CL received honoraria for travel expenses for attending meetings from Genzyme and Roche. MS received research support and speaking honoraria from Biogen, Merck, Roche, Sanofi, and Novartis. SZ received fees for travel expenses for attending meeting and consultation from Novartis.

The remaining authors declare that the research was conducted in the absence of any commercial or financial relationships that could be construed as a potential conflict of interest.
